# Hand size underestimation grows during childhood

**DOI:** 10.1038/s41598-019-49500-7

**Published:** 2019-09-13

**Authors:** Lucilla Cardinali, Andrea Serino, Monica Gori

**Affiliations:** 1 Fondazione Istituto Italiano di Tecnologia, Genova, Italy; 2 MySpace Lab, Department of Clinical Neuroscience, Centre Hospitalier Universitaire Vaudois (CHUV), University of Lausanne, Lausanne, Switzerland

**Keywords:** Neuroscience, Psychology

## Abstract

Cortical body size representations are distorted in the adult, from low-level motor and sensory maps to higher levels multisensory and cognitive representations. Little is known about how such representations are built and evolve during infancy and childhood. Here we investigated how hand size is represented in typically developing children aged 6 to 10. Participants were asked to estimate their hand size using two different sensory modalities (visual or haptic). We found a distortion (underestimation) already present in the youngest children. Crucially, such distortion increases with age and regardless of the sensory modality used to access the representation. Finally, underestimation is specific for the body as no bias was found for object estimation. This study suggests that the brain does not keep up with the natural body growth. However, since motor behavior nor perception were impaired, the distortion seems functional and/or compensated for, for proper interaction with the external environment.

## Introduction

In the first 10 years of life, our body goes through an impressive amount of changes in size, shape, weight distribution, capabilities and so on. While each physical conquest brings new opportunities of interaction with the environment, it also constitutes a challenge for the brain. For example, by 7 months of age, babies back and neck muscles are strong enough to allow them to sit unsupported^1,2^. This allows gaining a different perspective on the world and frees the hands for exploration. Better object manipulations abilities require and trigger new patterns of fingers behaviors, which, in turn, leads to richer exploration and interaction with the environment. Similarly, by two years a child can use objects while holding them and develops blended grasping patterns allowing to move an object while maintaining a grasp on it. The relationship between object manipulation and motor skills such as locomotion is even more complex and bideractional, as shown by Karasik and colleagues^3,4^. Indeed, they showed that walkers tend to act on a larger space, carrying objects and sharing them more with other people, compared to crawlers. At the same time, the type and amount of objects manipulation at an early age was predictive of transition to walking in crawlers. The increased amount of inputs from space requires better multisensory integration thus potentially triggering optimal integration. In other words, every change in body features originates a cascade of consequences at motor, perceptual and cognitive level^3^. Because of their pervasive effects, body changes need to be monitored and appropriately represented in the brain, especially during development.

Previous work showed how the brain is indeed capable of doing so, even at a very young age^5,6^. The first level of body representation explored by previous studies focused on the integration of proprioceptive/postural and tactile bodily cues. Such ability seems to develop quite early, as changes in body postures have been shown to affect tactile discrimination in babies as young as 8 months^7,8^. Studies on older children provided information on how the brain is capable of efficient online arm and hand position tracking^9,10^ to serve efficient motor control. Finally, a higher level of body representations, such as the ones supporting the sense of self and body ownership has been proven plastic enough to incorporate external objects at a very early age and distinguish between self and others^11^.

While covering several aspects of body representations, the available literature on children is quite spare and fragmented. On one hand, it mainly focuses on body posture, almost entirely ignoring body size representation, despite it changes the most in children and is critical for motor control and behavior. One exception worth mention here is the well-known Scale Error shown by young children up to 30 months of age. These children would typically try wearing dolls cloths that are clearly too small for them or entering toy cars they can not fit in. It has been proposed that they rely on a body representation that is not capable to fully account for the actual growth. Interestingly, at the same age children show error in estimating non-bodily stimuli too. However, Brownell *et al*.^12^ found that children’s performance on tasks that required them to reason about relative object size was unrelated to their performance on tasks that required the use of body size knowledge. The authors suggested that this support the existence of different mechanisms to represent body size and objects size. This dissociation might be experience driven and serving an important role in action performance: indeed, as we continuously act on different objects, precise and rapid size coding for objects is fundamental for acting, while the time scale of body size changing is different and slower^13,14^.

Based on these results we can make the hypothesis that children will show differences when estimating their own hand vs. an object. In particular, we expect to find smaller errors in objects size estimation compared to hand estimation.

On the other hand, previous studies usually do not cover an extended period of development, but rather focus on a specific age, which provides still pictures of body representations development at a specific age, with no insight on its dynamics, i.e., on the years preceding or following the time of the investigation.

**Figure 1:**
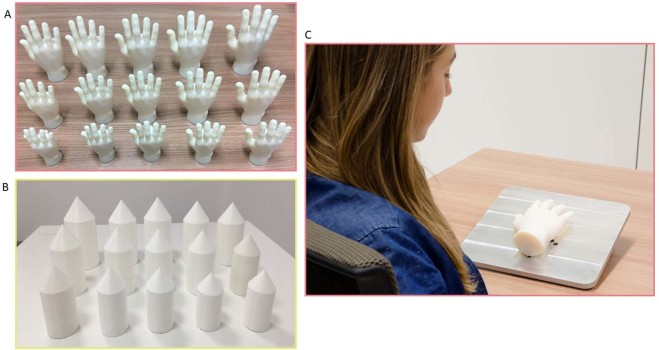
3D printed hands and objects used for Experiment 1 and 2 respectively. (**A**) 15 different 3D printed Hands used in Experiment 1. (**B**) 15 3D printed objects used in Experiment 2. (**C**) In Experiment 1, participants were presented with one hand at a time and asked to judge whether it was smaller or larger than their own. In Experiment 2 participant were presented with a reference object on the right space, either in front of them (in the Visual condition) or under their right hand (haptic condition). At each trial, a second target object was presented to the left and participant was asked to judge whether the target was smaller or larger than the reference.

In this paper, we make a first step toward overcoming both limitations by (1) focusing on hand size, in a large group of children (N = 84), (2) tested along an age range spanning between the 6 and 10 years. The age range was chosen for two reasons: first, as we were introducing a new task, we wanted to be sure that participants could clearly understand instructions and perform the task. Second, previous studies from our group^15,16^ showed that children rely on visual or haptic information differently, depending on the object feature they have to judge (size or orientation) and they do not reach optimal multisensory integration until 8 years of age. This study is a first investigation of hand size representation in children using visual and haptic information; hence, we decided to sample participants of an age range cantered around 8. Among all body parts, hands have a very important and peculiar role as privileged mean of interaction with the environment while being the main source of tactile information. Because of this important role, one might think that our brain contains an extremely precise representation of our hands. This has been empirically proved not to be true: distortions are present at every level of hands representation in adults^17^, starting from primary somatosensory and motor maps (homunculi) which do not represent veridical body size but rather a functional size, to a higher level, consciously accessible representations. Since the pervasiveness of distortions in body representation in adults, here we investigated whether they are present in children to assess whether body representations are always distorted or distortions emerge at a particular age. We presented children with 3D printed human hands of different size and ask them to judge whether each of them was bigger or smaller than their own hand (Fig. 1). Using the method of limits, we presented hands in 10 consecutive series, alternating series where hand size progressively increased to series in which size decreased. In addition, we also tested the role of different sensory modalities in the perception of the hand size by asking children to estimate their hand using selectively either visual or haptic information (via manipulation while blindfolded). Finally, to assess whether size judgments bias is body specific, we performed a control experiment asking the same children to judge the size of a non-bodily object (3D printed pointed cones, matching the printed hands size), via either visual or haptic exploration.

## Results

For each child, we calculated the amount of size distortion (real - estimated hand/object size) in the visual and haptic condition for both experiments (Exp. 1: Hand size estimation and Exp. 2: Object Size estimation). In order to assess both the precision and the coherence of the judgements, we then ran two separated ANOVAs, one on the average and one on the variability (standard deviation) of the distortion values across the 10 series of stimuli presentation, with the within-subjects factors Task (Hand vs. Object) and Modality (Visual vs. Haptic) and Age Group (5 levels, 6 to 10 years) and Gender as between-subjects factors. For significant interactions, we conducted post hoc analysis using t-tests for independent samples with unequal variance applying Bonferroni correction for multiple comparisons.

### Size underestimation (average)

**Figure 2:**
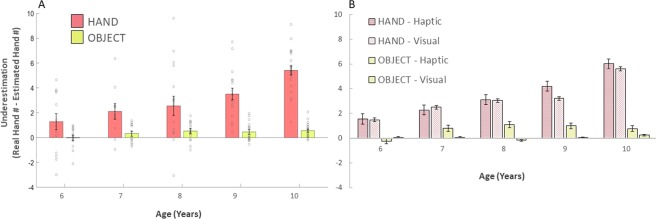
Hand and Object size estimation across time for the Haptic and Visual Condition. (**A**) Children significantly underestimate their hand size while being accurate in estimating an object size. While object size estimation remains consistently accurate across time, hand underestimation increases during childhood with older children underestimating their hand size more than older children do. Bars represent group values. Dots represent single subjects’ values. (**B**) Children underestimate their hand in both visual and haptic task. Error bars indicate standard error.

The first ANOVA returned a main effect of Task (F(1,83) = 90.618, p < 0.01; η² = 0.472) showing that children misrepresented their hand, but not the object size (Fig. 2A). In particular, the direction of the estimation error showed that children underestimated the size of their hand, but were correct when they had to judge an object. We also found a main effect of Age (F_(1,83)_ = 7.184, p < 0.01; η² = 0.273). Post hoc analysis revealed higher underestimation for the older children (10 and 9 years old) compared to the youngest (6 years old) and the 8 years old group (all p < 0.01; Fig. 2A). Importantly, this effect was further characterized by a significant Experiment*Age interaction (F_(4,83)_ = 36.625, p < 0.01; η² = 0.154; Table 1), showing that older children underestimated more their hand compared to younger ones, whereas their judgments of objects size remain stable and correct. No effect of Gender (F_(1,83)_ = 1.937, p = 0.115; η² = 0.074), nor interactions with Gender were also found (all p values > 0.08).

**Table 1:** ANOVAs post hoc tests p-values for the Experiment*Age interaction.

Age Groups	6 years	7 years	8 years	9 years	10 years
6 years	0.021*	0.20	0.04*	<0.01*	0.00*
7 years		0.03*	0.29	0.09	0.01*
8 years			<0.01*	0.22	<0.01*
9 years				<0.001*	<0.001*
10 years					<0.001*

White cells shows values for the Age Groups comparisons. On the diagonal (underlined text) values for the Experiment 1 vs. Experiment 2 comparison for the Age Group indicated in the column label.

**Figure 3:**
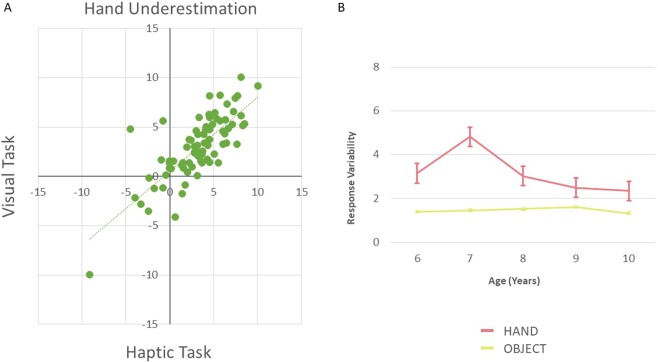
(**A**) Correlation between Visual and Haptic task for Experiment 1. (**B**) Hand and Object size estimation variability. Children show higher variability when judging their hand compared to judging an object size.

These effects appeared equally present for both modalities of testing, as we did not find any significant effect of Modality (F_(1,83)_ = 3.876, p = 0.053; η² = 0.045; Fig. 3), nor any interaction with Modality (all p values >0.12). While the Haptic vs. Visual comparison was not significant, it is worth mentioning a trend, for the hand Task toward a higher underestimation in the haptic condition (compared to the visual one) in older children (9/10 years old; Fig. 2B). This might suggest that younger children rely on a single representation and only with age develop separate ones that are accessed by and based on different sensory modalities. To further investigate this aspect and assess whether the two modalities (visual and haptic) tap into the same representation or two (equally distorted) different ones, we computed a Pearson correlation between the amount of underestimation in the Visual and Haptic modalities. We found a significant correlation (r = 0.776, p < 0.001), suggesting that both modalities accessed the same representation (Fig. 3A).

### Size underestimation (variability)

We run a second ANOVA to assess any difference in the variability of the participants’ answers across the 10 judgment repetitions. We found a main effect of Task (F_(1,83)_ = 48.071, p < 0.01; η² = 0.39), Modality (F_(1,83)_ = 68.032, p < 0.01; η² = 0.468) and an interaction of the two (F_(1,83)_ = 16.192, p < 0.01; η² = 0.18). As shown in Fig. 3B, responses variability was higher for judging the hand size compared to the object and when such judgments were made based on haptic information rather than visual ones. Interestingly, the interaction showed that variability was lower for the visual modality as compared to the haptics modality for the Object experiment, suggesting higher precision for visual estimation. However, this was not the case for the Hand experiment, whereby variability was comparable (and higher than the object condition) for both modalities, showing a general reduction of precision for bodily stimuli, independently from the modality of test. These effects were constant across age and gender, as there were no main effects, nor interactions with these variables (all p-values > 0.08). Given that accuracy for hand estimation varies with age, while variability differs only for hand/object conditions, higher hand estimation in older children cannot depend on changes in variability.

## Discussion

The goal of the study was to investigate a rather neglected feature of body representation, i.e., hand size perception, in a large sample of young children, from 6 to 10 years old.

We found that children underestimate the size of their hands, while being accurate in judging objects size, a bias mirroring hand perception in adults^11^. This was true independently on the sensory modality, as underestimation was equally present for visual and haptic task, whenever it involved hands perception. Most importantly, the amount of underestimation increased with age, with younger children being relatively accurate compared to older children. In other words, it looks like the hand representation in the brain cannot keep up with the actual hand growth. Crucially, the increase of the underestimation was not the result of an increase in uncertainty in the response as older children less variability in their responses while being less accurate in the representation of their hand size.

Our data show that size underestimation emerges as a characteristic of body representations since a very young age. The question is why our brain represents, at a conscious level, our hands as smaller than they are.

One explanation could be in the over-representation in primary somatosensory and motor maps and the need for a compensation for it. Indeed, in both homunculi, hands occupy a very large cortical territory compared to other body parts, despite their physical size. However, this overrepresentation is not accompanied by biases in behaviour, as one would expect if such maps were used to control movements or organize perception. If we were to plan movements according to hand size as represented in M1 we would exhibit grasping errors.

Similarly, in the Weber Illusion a same distance between two tactile stimuli is perceived differently depending on whether the two stimuli are delivered on body part with lower or higher tactile acuity. For instance, two touches delivered on the fingers will be perceived as further apart as compared to the same touches delivered at the same distance from each other on the back. However, the hand over-representation in the primary somatosensory cortex can only partially explain this illusion^18^, as a much stronger bias would be predicted on the basis of the cortical magnification factor between different body part, which suggests the presence of a compensatory mechanism at a higher level. This hypothesis is supported by another perceptual effects whereby tactile distance perception is influenced by magnified or minified vision of the stimulated body part^19^, or illusory change in body part size as induced by proprioceptive stimulation^20^. Thus, it seems as if, in an attempt to compensate for a bias at the primary level, the brain exceeds on the opposite direction which results in an underestimation at a higher level of representation. The question then is why perception biases go in the direction of an underestimation.

Previous studies have shown how body representations at a different level are more prone to modifications in the direction of an extension/elongation, rather than the opposite (see van der Hoort & Ehrsson for different results). For example, Pavani and colleagues showed, using a Rubber Hand Illusion paradigm, how hands larger than the real one are easier to incorporate than smaller ones^21^. Similarly, body representations have been shown to be capable of incorporating long tools, elongating the arm length representation^22,23^, while short-term manipulation difficulty induce a perceive contraction^24^. One can make the hypothesis that given this directional advantage, a representation that underestimates can be quickly compensated when needed. However, this does not explain why non-veridical representations are present by default nor whether underestimation produces the elongation bias or vice versa.

Another hypothesis is that the underestimation is a more complex distortion that reflects multiple compensatory mechanisms. Since data on body size representations in children are not available, once again we can refer to the adult literature, which is richer. Hand representations are distorted in young adults and, in particular, we tend to represent our hands not simply as smaller^25–27^: fingers length is underestimated but not uniformly as the underestimation increases as we move from the thumb to the little finger. Meanwhile, knuckles distance is overestimated leading to a represented hand with short and more distant fingers than reality. This suggests that, if the underestimation is the result of an overcompensation the brains applies to the primary sensory and motor representations, this does not happen uniformly. It seems that less compensation is applied to the fingers that contribute the most to our dexterities, suggesting a role of motor experience in this dynamics.

Previous studies already showed how motor and sensory experience induce changes in body representation organization: for example, in the first months of life babies rely on canonical body representations for posture where, for example, the right hand occupies the right space and vice-versa for the left^7,28^.When the baby begins showing spontaneous reaches across the midline, behaviours such as the cross-hands effect, resulting from updated arm posture representation, start to appear. Similarly, one can make the hypothesis that the amount of sensory inputs received and motor skilfulness acquired with the hands affect their size perception.

Our data add a fundamental and contradictory new piece of information: we showed that underestimation increases with age, meaning that distortion increases with the acquisition of skillful motor control^29^. If we compare the amount of underestimation in the present sample of children and the adult literature^30^, we can see that the amount of underestimation is even higher from childhood to adulthood. This comparison has to be taken with caution because based on data from different tasks. One important difference between our task and Longo and Haggards’ is that we assess size estimation globally, while they investigated hand’s segments. It is possible that the global reduction in hand size estimation in children is the result of a distortion where fingers and palms are differently affected. What we capture here is a hand representation that seems not capable of following the entire hand in its global growth, although given the cross-sectional nature of our study we can not make strong inferences about the development of the hand representation but rather present snapshots of its state at different ages. Future research might focus on the development, using a longitudinal approach and studying single digits representations.

Another crucial result of our study is that hand distortion was present both for the visual and haptic condition. Previous studies on adults have proposed the existence of multiple body representations, based on the observation of differences in the amount and/or quality of the distortions in body representations and the sensory information they rely on (somatosensory vs. visual^31–35^). The debate about whether these representations are completely independent or interconnected is still ongoing, which makes relevant any study showing evidence in favor or contrary to a dissociation between vision-based and somatosensory-based representations^33^. Here we found a similar distortion (underestimation) in both visual and haptic tasks, a result that seems to contradict recently proposed theories according to which visual and somatosensory representations both show distortions, but in opposite directions to properly compensate for each other’s biases^36^. Our results show that such difference is not present when children judge hand size. Thus, they suggest that the hand size perception task employed here taps into the same (distorted) representation, that is accessible from the visual or somatosensory modalities, at least in children. The presence of a strong correlation between our two tasks results seem to exclude the alternative possibilities that two different representations are stored and used to judge hand size in either modality and both are equally distorted.

Finally, we showed that the distortions involve only hands’ size and not objects’, even when hands are used to create that judgement (that is, in the haptic condition). This points to a body specific distortion, in line with previous studies on Scale Errors on younger children. However we must consider a crucial difference between the two Experiments: while both required a comparison between a standard stimulus that is always present (Own hand in Experiment 1 and Right object in Experiment 2), the presentation modality of such standard stimulus differed, especially in the visual condition. By being attached to the rest of the body, information from the hand are always available through somatosensation, making the haptic condition quite similar between Experiment 1 and 2. This is not the case for the visual condition, in which we prevented children from seeing their own hand and forced them to rely on storage knowledge. This could suggest that the nature of the task (off-line in Experiment 1 and On-line in Experiment 2) could play a role in explaining our results. While we can not completely exclude this hypothesis, since we did not find any difference between the two conditions in both Experiments, we are quite confident that Experiment 2 constitutes a valuable control and supports our interpretation of a body-specific bias.

In conclusion, we showed, for the first time, that hand representation in children ages 6 to 10 is distorted and in particular that similarly to adults, children underestimate their hand’s size. Crucially the underestimation increases with age, is specific for the hand (as it is not present when judging an object size) and independent on the sensory modality provided.

These data provide a first insight into the development of body representations in childhood and show the appearance of a progressive divergence between the actual growth and the represented growth at a global level.

## Methods

84 children (age range 6–10 years old; 37 Females, 47 Males, mean age 8.13 yo, SD 1.46) were recruited for the study through a local school. Children were divided in age groups (6 years old: N = 19; 7 yo: N = 15; 8 yo: N = 15; 9 yo: N = 20; 10 yo: N = 15) Informed consent was obtained from the parents before testing. Experiments were approved by the internal ethical committee (Comitato Etico, Asl 3, Genova, protocol name: IIT_COMP_MIS) and conducted in accordance with the principles of the revised Helsinki Declaration (World Medical Association, 2013).

The study was composed by two experiments during which participants were asked to estimate the size of their own hand (Experiment 1 – Hand) or of an object (Experiment 2 – Object).

Each experiment took approximately 30 minutes and children were told they could take breaks or interrupt the experiment at any moment.

Experiment 1. The experiment took place in a familiar environment (one of the school room). Children were asked to sit at a desk in front of a metal support on top of which one of the 15 3D printed right hands was presented at each trial. The plastic hands were 3D printed at the Italian Institute of Technology (IIT) using a Stratasys Objet Connex 500 with a PolyJet Technology. The hands differed in size only. Measurements, shown in Table 2, were chosen after measuring hand length and width in an independent group of 30 children aged 4 to 12 years old. Between one hand and the next one on the scale, there was a 0.5 cm increment in length while width incremented by 0.5 cm every two hands. This was done to maintain a Length/Width ratio between 1.9 and 2, which appeared to be the most prominent feature, based on our hand size survey.

**Table 2:** 3D printed Hands and Objects size measures.

Hand/Object #	Length (cm)	Width/Diameter (cm)
1	9.5	5
2	10	5
3	10.5	5.5
4	11	5.5
5	11.5	6
6	12	6
7	12.5	6.5
8	13	6.5
9	13.5	7
10	14	7
11	14.5	7.5
12	15	7.5
13	15.5	8
14	16	8
15	16.5	8.5

The size estimation task was performed in two different modalities, in two separate blocks: a Hand Visual estimation condition (Hand - V) and a Hand Haptic estimation condition (Hand - H). Blocks order was counterbalanced across participants.

In the Visual Condition (Hand - V), participants were asked to hide both hands behind their back or in their pockets for the entire duration of the block. Then the experimenter asked the child to close his/her eyes while placing the first plastic hand on the metal support. The child was then allowed to open his/her eyes and look at the plastic hand for as long as he/she wanted before telling the experimenter if his/her own hand was larger or smaller than the seen one. Once the judgement was made, he/she was asked to close the eyes again while the experimenter changed the plastic hand in the display. Hands were presented in 10 consecutive decreasing or increasing series. The first two series always started from the two extreme sizes (Hand #1 and Hand #15), in a counterbalanced order across participants. Starting from the third series, when a change in judgment happened (transition point), the experimenter chose the start of the following series 4 or 5 steps away in the opposite direction. This was done to reduce testing time and at the same time to avoid making the starting point predictable.

In the Haptic Condition (Hand - H), stimuli presentation followed the same procedure, but children were blindfolded and were allowed to explore the plastic hand using their left hand only while the right one rested on their lap. Participants were left free to explore the plastic hand as they wanted and for as long as they wanted before giving their answer. To calculate size underestimation we first identified, for each child, the fake hand which size was equal to his/her real one. Then we calculated the difference between the number of that hand and the average of the transition points across the 10 repetitions.

Experiment 2. This experiment was identical to Experiment 1 except for the fact that children were asked to estimate the size of an external object. Object was 3D printed cylinder with a pointy tip. Their size (height and diameter) matched one of the 15 plastic hands. For each participant, the target object to be estimated (see below) was the one whose size matched best the size of their real hand.

In the Visual condition (Object – V), participants were presented with a first object, placed on the table in front of them slightly to the right of their midline, which remained the same for the entire block (target object). At the beginning of each trial, the child was asked to close the eyes while a second object was placed next to the first one, on the left. The child was then asked to open the eyes and observe the objects before saying if the one on the right (target) was larger or smaller than the one on the left. Once the judgement made, the child was asked to close the eyes again while the object on the left was switched with another one following the same procedure described above for the plastic hands. 10 consecutive series were presented, alternating increasing and decreasing ones.

For the Haptic condition (Object – H), participants were blindfolded. The target object was placed horizontally on the table under the right hand. The same object was used for the entire block and never changed. However, every few trials, children were encouraged to move their right hand to avoid habituation and reduction of sensitivity. On each trial, a different object was placed under the left hand and children were left free to explore it haptically as they wanted and for as long as they wanted before giving their response (“larger” or “smaller”).

10 consecutive series were presented, alternating increasing and decreasing ones.

The order of the two blocks (Visual and Haptic) was counterbalanced across participants. Similarly to Exp. 1, object underestimation was calculated as the difference between the reference object number and the average transition point across the 10 repetitions.
